# Trends in the Prevalence of Overweight, Obesity and Severe Obesity in Primary School Children in Croatia from 2003 to 2022

**DOI:** 10.3390/children12101299

**Published:** 2025-09-25

**Authors:** Sanja Musić Milanović, Lucija Sironić, Ana Pezo, Helena Križan, Vesna Jureša, Vera Musil

**Affiliations:** 1 Croatian Institute of Public Health, Division for Health Promotion, 10000 Zagreb, Croatia; sanja.music@hzjz.hr (S.M.M.);; 2 School of Public Health “Andrija Štampar”, University of Zagreb School of Medicine, 10000 Zagreb, Croatia; 3 Teaching Institute of Public Health “Andrija Štampar”, 10000 Zagreb, Croatia; 4 Institute of Public Health of Zagreb County, 10290 Zaprešić, Croatia

**Keywords:** overweight, obesity, severe obesity, prevalence, children, Croatia

## Abstract

**Highlights:**

**What are the main findings?**

- The prevalence of overweight (including obesity) steadily increased over time, particularly until the mid-2010s, whereas the prevalence of severe obesity had the opposite trend throughout.
- The lowest prevalence rates of overweight and obesity were observed in the capital, with other regions exhibiting significantly higher risks.

**What is the implication of the main finding?**

- Despite a slowdown in the growth of overweight (including obesity) prevalence in recent years, Croatia has yet to reach the plateau observed in some other European countries.
- Regional differences in the overweight and obesity risk require a follow-up.

**Abstract:**

**Background:** Childhood obesity remains a significant public health challenge globally. Croatia ranks among the leading European countries in terms of childhood overweight and obesity prevalence. **Methods:** The present cross-sectional study analysed overweight and obesity prevalence trends from 2003 to 2022 on a nationally representative sample containing data from five studies, two independent studies and three WHO COSI rounds. Data from a total of 11,817 children aged 7.00–8.99 were analysed. Weight categories were defined using the IOTF and WHO cut-offs. Overweight and obesity prevalence rates and trends overall and by sex and region were calculated, and binary regression models applied to investigate the relationship between the risk of overweight and several variables. *p*-value < 0.05 was used to define statistical significance. **Results:** Temporal trends and associations were investigated using the IOTF reference values. The prevalence of overweight (including obesity) had increased steadily from 2003 to 2015, thereafter continuing to increase at a slower rate, whereas the prevalence of severe obesity reduced over time. Even though boys had slightly higher prevalence rates of overweight, the growth in overweight prevalence in girls over time was significant. At the regional level, the lowest prevalence rates were detected in the capital (City of Zagreb region). The risk of overweight was at least 50% higher in all the other regions in Croatia, and a rising trend in overweight risk with time was particularly high among children in the Adriatic and Northern regions. **Conclusions:** Despite a deceleration in the rate of increase in overweight (including obesity) prevalence, Croatia is yet to reach a plateau observed in some other European countries. Unearthed regional differences warrant further investigation.

## 1. Introduction

Obesity is a complex chronic disease known to negatively impact individual’s lifespan and healthspan [1] and is a recognised risk factor for several non-communicable diseases [2]. It is a strong predictor of overall mortality, several cause-specific mortalities and reduced quality of life [1,3]. The burden of chronic disease risk is already increased at pre-obesity (overweight) stage [4]. Given the major implications of obesity and overweight on both individual- and population-level health, as well as high economic costs that management of these carries [5], it is essential that obesity and related clinical phenomena are closely monitored, and that their prevention and treatment are invested in early in life.

Among children, the burden and prevalence of overweight and obesity have continuously increased globally over the past five decades, having grown from an international epidemic [6] into a pandemic [7]. In 2022, every fifth child in the world had overweight or obesity, which represents a 2.5-fold increase in overweight prevalence from 1990 [8]. Future global estimates expect both overweight and obesity rates to continue to rise so that by 2035 almost 40% of children globally may not be of healthy weight [9]. Also worrying is the increasing prevalence of severe obesity [10]. Obesity in childhood has significant ramifications on psychosocial and physical health, function and participation beyond childhood [6,10]. Early-onset and more severe obesity may reduce life expectancy by decades [11] and lead to more pronounced comorbidities [12].

In Europe, Croatia is ranked first, alongside Malta, in the prevalence of overweight and obesity in adults, with two thirds of its adult population having overweight or obesity according to the latest European Health Interview Survey (EHIS) [13]. Childhood overweight and obesity pose yet another challenge to the Croatian healthcare system. According to the latest WHO European Child Obesity Surveillance Initiative (COSI), the largest surveillance initiative of its kind globally [14], a third of all children in Croatia aged 8 to 9 years live with overweight or obesity [15]. This places Croatia amongst the frontrunner countries of the WHO European Region in the prevalence of overweight and obesity in school-aged children, alongside other Mediterranean countries such as Cyprus, Greece, Italy and Spain [15]. In the WHO European Region, the prevalence of overweight and obesity in children and adolescents aged 5 to 19 has been on a steady rise since 1975, particularly in the decade leading up to 2016 when the prevalence of obesity increased by 40% and that of overweight by 20% [14]. In Croatia, the trends of childhood overweight, obesity and severe obesity have not been described to date.

Despite concerning statistics, Croatia does not have a longitudinal anthropometric data collection programme in place for any age group, including school-aged children. Instead, it has historically relied on anthropometric data gathering in the form of cross-sectional surveys, which the present study draws upon. Fostering research quality around childhood overweight and obesity is, nonetheless, a prerequisite for the implementation of nation-level prevention plans and development of obesity management guidelines and strategies, all of which were highlighted as important areas for action in the EU Action Plan on Childhood Obesity in 2014 [16] and the national Action Plan for Obesity Prevention 2024–2027 [17]. The aim of the present study was to evaluate temporal trends in the prevalence of overweight, obesity and severe obesity in primary school children in Croatia aged 7.00–8.99 years, using the data collected as part of two independent studies [18,19] and three rounds of the COSI [15,20,21], covering a period from 2003 to 2022.

## 2. Materials and Methods

### 2.1. Study Participants and Data Sources

The present work is a cross-sectional study based on five datasets. Two originate from independent studies run between 2003 and 2008, namely School Health Survey 2003–2004 [18] and The Cardiovascular Risk Factors in School Age—Intervention Model Development 2006–2008 [19]. Both studies gathered anthropometric data (body weight, body height) from children in several school grades, including the first grade of primary school, with the target age group being 6.50–7.49 years. The remaining data included in the present study were collected during the three rounds of COSI between 2015 and 2022 [15,20,21]. The COSI rounds in Croatia, referred to as CroCOSI, collected anthropometric data in children attending the second and third grades of primary school, with the target age group being 8.00–8.99 years. From the five studies, anthropometric data on children aged 7.00–8.99 years (age in years rounded to two decimal places) were extracted and analysed, as were data on participants’ geographical distribution at level 2 of the Nomenclature of Territorial Units for Statistics (NUTS-2). The study included only participants with complete records, defined by the presence of a data collection timepoint, recorded body weight and height, date of birth, sex and region. More information on the descriptive characteristics of the present study and its participants may be found in Table 1. All five studies used standardised instruments and techniques to obtain children’s anthropometric measures. Participating schools, that is, their classes, were randomly selected from a pool of schools and classes obtained from the Ministry of Science, Education and Youth of the Republic of Croatia. In the first two studies [18,19], the primary sampling unit was school, and the secondary sampling unit was school class. The primary sampling unit in CroCOSI studies was class [15,20,21]. Data collection procedures for each study are described in more detail elsewhere [15,18,19,20,21].

Croatia joined COSI in the school year 2015/2016 [21] and took part in the following two rounds run in 2018/2019 [20] and 2021/2022 [15]. COSI, established in 2006 in response to a growing issue of childhood obesity in the WHO European Region, measures standardised weight and height on nationally representative samples of primary school children between the ages of 6 and 9, and gathers information on a number of factors known to contribute to overweight and obesity in children [22], namely dietary habits, physical activity level, socioeconomic status, school environment, screen time and sleep duration [23,24,25].

### 2.2. Definition of Overweight, Obesity and Severe Obesity

To maximise comparability to other research work, weight categories (overweight, obesity and severe obesity) were defined according to both the International Obesity Task Force (IOTF) [26,27] and the WHO cut-off points [28]. These two growth references are commonly used in epidemiological research, although they yield differing prevalence estimates owing to different reference populations and methodologies. Prevalence rates tend to be higher when applying the WHO growth reference [29]. Recently, a harmonisation algorithm has been proposed allowing for conversion of prevalence estimates between the two systems [30]. Overall prevalence rates, and prevalence rates by sex and region were calculated using both BMI reference systems. Prevalence trends and statistical models used the IOTF cut-offs only. Importantly, the estimates of overweight prevalence included combined prevalence of overweight and obesity (hereafter *overweight (including obesity)*), and the estimates of obesity prevalence included combined prevalence of obesity and severe obesity.

### 2.3. Data Processing and Statistical Analysis

Each study dataset was reviewed for inconsistencies and completeness prior to analysis. Descriptive analysis included estimation of overweight, obesity and severe obesity prevalence rates and trends over five timepoints overall, by sex and by NUTS-2 region. Here, time was treated as a categorical variable. Prevalence estimates were presented as percentages. Prevalence trends were assessed by linear-by-linear association chi-square (χ^2^) test. *p*-value < 0.05 was used to define statistical significance.

Next, binary logistic regression models were applied to assess the relationship between the risk of overweight including obesity and independent variables, and between the risk of obesity and several variables. The first model included time (linear), sex, age (linear) and region as independent variables simultaneously. Next, separate models for either sex were run, adjusting for time (linear), age (linear) and region simultaneously. Finally, separate models for each region were run, adjusting for sex, time (linear) and age (linear) simultaneously. The data were analysed using SPSS software (version 25.0).

## 3. Results

The results herein presented are based on data from a nationally representative sample of 11,817 children aged 7.00–8.99 years, collected during five timepoints between 2003/2004 and 2021/2022. Table 1 summarises the study participants’ characteristics.

The prevalence rates of overweight, obesity and severe obesity over the five timepoints were calculated according to both the IOTF and WHO cut-offs for completion purposes and are showcased in Table 2. The data in this table clearly point to two BMI reference systems yielding significantly different prevalence rates, with rates based on the WHO growth reference being higher than those derived from the IOTF, consistent with findings in the literature [29]. Comparisons of prevalence rates by sex and region between the two BMI reference systems are available in the Supplementary Material (Supplementary Tables S1 and S2). To avoid confusion, the prevalence trends and results of the statistical models described in the text under subheadings 3.1–3.5 were obtained using the IOTF cut-offs only.

### 3.1. Prevalence Trends of Overweight, Obesity and Severe Obesity

The prevalence of overweight (including obesity) among Croatian school children aged 7.00 to 8.99 years was found to have continuously increased since 2003, as showcased in Table 2. The difference in prevalence rates was shown to be statistically significant (*p* = 0.000). The prevalence of obesity largely followed this trend, except for the last, fifth round of measurement when a slight decrease in obesity prevalence was observed (*p* = 0.484). Severe obesity demonstrated a downtrending prevalence, but the changes were not statistically significant (*p* = 0.137).

### 3.2. Overweight, Obesity and Severe Obesity Prevalence by Sex

**Figure 1:**
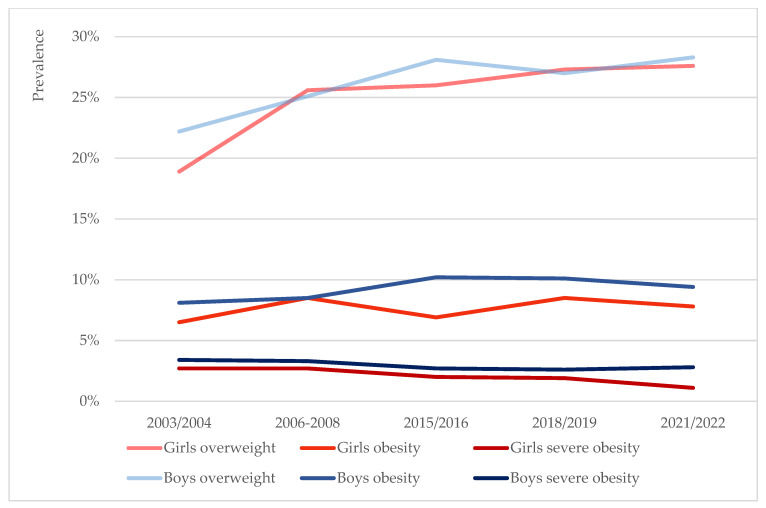
Prevalence trends of overweight, obesity and severe obesity by sex based on the IOTF growth reference.

The prevalence of overweight (including obesity) was observed to be overall higher among boys (26.9%) than girls (26.1%) for the duration of the study. In both sexes, the prevalence of overweight saw an increase over time (Figure 1). In boys, the prevalence increased from 22.2% in 2003/2004 to 28.3% in 2021/2022 (*p* = 0.048), and apart from a slight decrease between the 2015/2016 and 2018/2019 timepoints, it was overall uptrending. In girls, the prevalence of overweight was relatively lower than in boys at start (18.9%), after which it saw a steady increase in subsequent rounds, so that by the last round of measurement it was almost on par with the overweight prevalence in boys (27.6%) (*p* = 0.005).

The prevalence of obesity was also observed to be overall higher among boys (9.6%) than girls (7.7%) for the duration of the study (Figure 1). In boys, it saw a peak in the third round of measurement, after which it started to decrease, though these changes were not found to be statistically significant (*p* = 0.415). In girls, the prevalence saw more fluctuations which were not statistically significant (*p* = 0.356).

Finally, severe obesity was again more common in boys (2.9%) than girls (2.0%) for the duration of the study. In girls, it was generally downtrending or stable: 2.7%, 2.7%, 2.0%, 1.9% and 1.1% in 2003/2004, 2006–2008, 2015/2016, 2018/2019 and 2021/2022, respectively (Figure 1). In boys, the prevalence was downtrending in all but the very last round of measurement: 3.4%, 3.3%, 2.7%, 2.6% and 2.8%, respectively. The observed changes in neither sex were, however, statistically significant (*p* = 0.781 for boys, *p* = 0.082 for girls).

### 3.3. Overweight, Obesity and Severe Obesity Prevalence by Region

**Figure 2:**
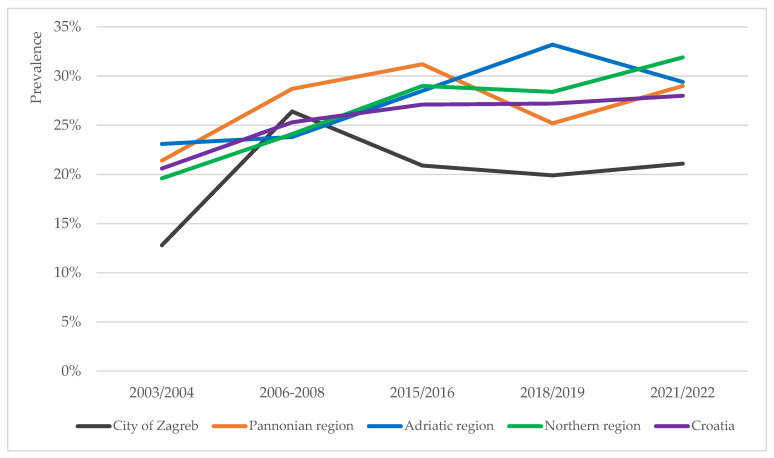
Prevalence trends of overweight (including obesity) across the four regions in Croatia (City of Zagreb, Pannonian, Adriatic and Northern regions) and national average, based on the IOTF growth reference.

**Figure 3:**
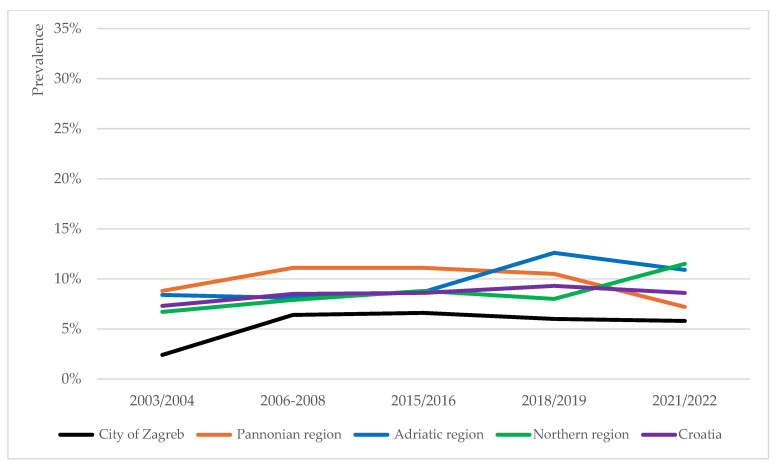
Prevalence trends of obesity across the four regions in Croatia (City of Zagreb, Pannonian, Adriatic and Northern regions) and national average, based on the IOTF growth reference.

**Figure 4:**
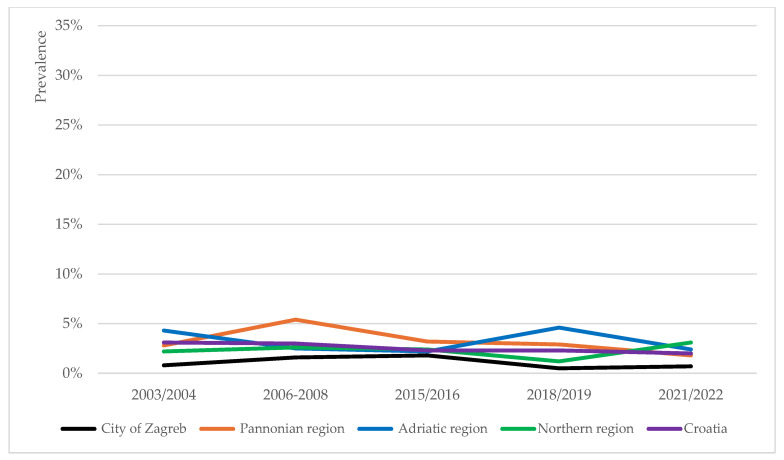
Prevalence trends of severe obesity across the four regions in Croatia (City of Zagreb, Pannonian, Adriatic and Northern regions) and national average, based on the IOTF growth reference.

The prevalence trends of overweight, obesity and severe obesity in school-aged children were analysed for four NUTS-2 regions in Croatia [31]: the Pannonian, Northern and Adriatic regions of Croatia, and the City of Zagreb. At the regional level, the prevalence trends of overweight (including obesity), obesity and severe obesity were of heterogenous nature (Figure 2, Figure 3 and Figure 4).

#### 3.3.1. Pannonian Region

The prevalence of overweight (including obesity) among children in the Pannonian region recorded the lowest rate in 2003/2004 and peaked in 2015/2016 (Figure 2) (*p* = 0.029). Changes in obesity prevalence in this region fit a U-curve, recording the lowest rate in the last timepoint (Figure 3) (*p* = 0.060). The prevalence of severe obesity in this region was higher than the national average for the most part (Figure 4), and the recorded fluctuations were statistically significant (*p* = 0.013).

#### 3.3.2. Northern Region

In the Northern region, the prevalence of overweight (including obesity), obesity and severe obesity all peaked in the last timepoint (Figure 2, Figure 3 and Figure 4). However, only changes in overweight (including obesity) rates were statistically significant (*p* = 0.006).

#### 3.3.3. Adriatic Region

In the Adriatic region, the prevalence rates of all three phenomena (overweight including obesity, obesity and severe obesity) peaked in the 2018/2019 timepoint, thereafter decreasing (Figure 2, Figure 3 and Figure 4). These three peaks mark the absolute highest prevalence rates of overweight (including obesity) (33.2%) and obesity (12.6%), and second highest prevalence rate of severe obesity (4.6%) detected at the regional level for the duration of the study. Changes in the prevalence rates were all statistically significant (*p* = 0.000, *p* = 0.023, and *p* = 0.021, respectively).

#### 3.3.4. City of Zagreb

In comparison to other regions, the City of Zagreb persistently recorded lower prevalence rates of overweight (including obesity), obesity and severe obesity for the duration of the study (Figure 2, Figure 3 and Figure 4). Only changes in the overweight prevalence rates were statistically significant (*p* = 0.042).

### 3.4. Overweight (Including Obesity) Risk Modelling

Binary logistic regression models revealed that NUTS-2 region and year of measurement had a statistically significant effect (both *p* = 0.000) on modelling the risk of overweight (including obesity) amongst the measured children. In essence, children were more likely to have overweight if they lived anywhere other than in the City of Zagreb (Table 3), with the highest risk recorded for those living in the Adriatic region (Table 3: OR 1.529). The risk of overweight also increased with time (Table 3: OR 1.017).

When the statistical model considered girls and boys separately, the risk of overweight (including obesity) in girls was highest if they lived in the Pannonian region (Table 4: OR 1.458). In boys, the risk was highest if they lived in the Adriatic region (Table 4: OR 1.620).

Accounting for region, the model found a very small but statistically significant increase in the risk of overweight (including obesity) over time only for children from the Adriatic (Table 5: OR 1.028) and Northern regions (Table 5: OR 1.027).

### 3.5. Obesity Risk Modelling

Binary logistic regression models looking into factors mediating obesity risk revealed similar findings to models describing the risk of overweight (including obesity). Again, living in any region other than the City of Zagreb increased the risk of obesity by 64% to 71% (*p* = 0.000). The risk was marginally higher as years progressed (*p* = 0.026) and lower for girls (*p* = 0.000) (Supplementary Table S3).

Stratified by sex, obesity risk in girls was highest if they lived in the Adriatic region (Supplementary Table S4: OR 1.835, 95% CI 1.359–2.479). In boys, it was highest if they lived in the Northern region (Supplementary Table S4: OR 1.613, 95% CI 1.231–2.112). These findings are not consistent with sex-stratified risk of overweight (including obesity), pointing out to different regions driving higher prevalence of obesity only in each sex. Additionally, risk of obesity was higher for boys at younger ages (*p* = 0.005) (Supplementary Table S4).

Accounting for region, the model found a very small but statistically significant increase in obesity risk over time for children from the Adriatic (OR 1.037, 95% CI 1.013–1.061) but not from the Northern region (Supplementary Table S5).

## 4. Discussion

The aim of the present study was to investigate the prevalence trends of overweight, obesity and severe obesity in Croatian school children aged 7.00–8.99 years, beginning with the earliest national data collected in 2003/2004. To our knowledge, there have previously been no attempts to characterise the trends in the prevalence of overweight, obesity and severe obesity in children of school age in Croatia.

The results demonstrate an increasing prevalence of overweight (including obesity) up until the mid-2010s, thereafter recording a slowdown in the growth rate. A plateau is yet to be reached, suggesting that Croatia is falling behind the trends in the prevalence of overweight (including obesity) of some European countries which have started to observe the overweight prevalence stabilising or even declining by the fourth round of the COSI [32]. It could be speculated that if Croatia is to follow the implementation of public health policies targeting the childhood overweight and obesity problem as successfully as some Southern European countries like Greece and Portugal that have halted or reduced their prevalence rates [32], it could similarly see the slowdown in the growth rate of overweight become a significant decrease in the subsequent rounds of the COSI. Nonetheless, it should be borne in mind that despite the decline in overall overweight prevalence, some countries have witnessed an increase in overweight prevalence in certain subgroups, for example, amongst children of lower socio-economic status (SES) [33]. Even with a fall in their prevalence, overweight and obesity remain a significant societal and economic public health challenge driving health inequalities [33].

### 4.1. Factors Affecting Overweight, Obesity and Severe Obesity Prevalence Trends in Croatia

No major differences in the trends of overweight (including obesity) prevalence between boys and girls were noted in the present study, which is line with other European countries [32]. Boys, however, maintained a higher prevalence rate of overweight by the last round of measurement. Based on the six COSI rounds run to date in the European Region of the WHO, boys have consistently demonstrated higher prevalence rates of overweight in comparison to girls [23,24,25].

The observed changes in obesity prevalence in primary school children in Croatia, though not statistically significant, demonstrate early signs of stabilisation and a potential for a decline in near future. Such a trend had previously been observed in some Mediterranean countries by the fourth round of the COSI [32]. As with overweight, obesity prevalence trends did not differ between girls and boys, as is the case in most European countries [23,24,25,32].

The prevalence of severe obesity has been declining on the national level, which is in line with the trend observed in some other European countries, such as Italy and Portugal, which share similar prevalence rates [34]. The prevalence was detected to be somewhat higher in boys than girls, which again matches the picture seen in other European countries [34].

Regional differences in the prevalence of overweight (including obesity) were, however, noted. As the economically most developed NUTS-2 region in Croatia [35], the City of Zagreb region recorded the lowest rates of overweight (including obesity) in the period from 2003 to 2022. Given the inverse relationship between overweight prevalence and gross domestic product (GDP) [36], and that between overweight prevalence and the level of urbanisation, particularly in high-income countries [37], Croatia included [38], this finding was not surprising. It was the Adriatic region that performed most poorly overall and recorded the highest prevalence rate of overweight (including obesity), as well as obesity and severe obesity. This finding conforms to and confirms the poor statistics of the Mediterranean parts of Europe to which the Adriatic region in Croatia belongs, and which lead the way in the overweight prevalence in children in Europe [23,24,25].

In the present study we show that children living in any region other than the City of Zagreb had a 48% to 53% higher risk of developing overweight (including obesity), and a 50% to 71% higher risk of developing obesity in the period from 2003 to 2022. Furthermore, our analyses reveal a rising trend in the risk of overweight (including obesity) over time among children living in the Northern and Adriatic regions, whilst the latter also recorded a higher risk of obesity only over time. Age, treated as a linear covariate, was not an independent predictor of overweight or obesity, except among boys in whom younger age was associated with higher risk of obesity only.

The regional differences examined here, based on the NUTS-2 division of Croatia into four regions, do not quite fit the geographical variations in overweight prevalence observed in other European countries, such as the north–south gradient observed in Italy [39] and Serbia [40]. They do, however, point in the direction of the urban–rural gradient having an impact on overweight and obesity prevalence, as witnessed in some other European countries including Serbia [40], Norway [41], Sweden [42] and Iceland [43] which recorded higher prevalence rates of childhood and adolescent overweight and obesity in rural areas. The results of this study are, nonetheless, perhaps most similar to what was observed in Hungary which recorded higher risk of obesity in all but one, the central region of the country, albeit only in boys [44]. What may be concluded from our work is that there is a so-called *capital city gradient* driving the risk of overweight, drawing considerable differences in the overweight prevalence and risk between the capital region of Croatia and other regions. Future studies should aim to understand which region-related factors (infrastructural, cultural, other environmental) play an important part in moderating the overweight risk and ideally discern if individual characteristics of measured children and their families are also important determinants [45].

### 4.2. Post-Pandemic Situation and Future Predictions

The study captured the data collected just before (the fourth timepoint, 2018/2019) and right towards the end of the COVID-19 pandemic (the fifth timepoint, 2021/2022). As per the results of a recent meta-analysis [46], there is evidence of an increase in BMI and obesity prevalence in children and adolescents during the pandemic, though such evidence has been deemed low certainty [46]. In our study, an increase in the prevalence of overweight but not obesity was in fact recorded between the 2018/2019 and 2021/2022 timepoints, albeit such a change was not statistically significant.

The upward trend in the overweight prevalence in children and adolescents up to 20 years of age in Croatia is expected to continue in the next few decades. Some predict that by 2060, a 130% increase in the overweight prevalence in boys in comparison to 2019 may be observed, meaning that nine in 10 boys will have overweight in comparison to four in 10 in 2019 [38]. In girls, the predicted increase of 193% in the overweight prevalence is even more drastic; this will see eight in 10 girls in this age group having overweight by 2060 in comparison to three in 10 in 2019 [38]. Overweight is projected to reduce life expectancy in Croatia by an average of 3.5 years over the period between 2020 and 2050, placing an even bigger burden on the national economy in the coming years [47].

### 4.3. Strengths and Limitations of the Study

The present study has several strengths. Firstly, the anthropometric parameters were obtained through direct measurements rather than self-reports, ensuring data reliability. Secondly, the overall sample size gathered over five timepoints was reasonably large, with almost 12,000 children included in the study. Additionally, all five studies included in the present study reported high participation rates (see Table 1). Finally, the present study collected and analysed data at the regional (NUTS-2) level, revealing interesting geographical–epidemiological disparities in a relatively small country, both area- and population-wise.

The limitations of the study are also not to be neglected. Socioeconomic data and measures of inequality were unfortunately not made available at the time of data analysis. These are extremely important if we are to understand the social gradient driving profound differences in overweight prevalence across the four regions. Furthermore, since the study compiled data from three COSI rounds and two inter-related independent studies, there were inevitably going to be differences in cohort sizes and participant age distribution between the datasets (Table 1). Most participants in the first study were 7-year-olds [18]. The split between 7- and 8-year-olds was almost even in the second study [19], whereas the COSI rounds recruited mostly 8-year-olds as this age group was their target [15,20,21]. Participant age distribution is a relevant factor, given that in other European countries, the prevalence of overweight (including obesity) was found to increase gradually with age between 6 and 9 years, in both sexes [48]. In addition, the COSI cohorts [15,20,21] were larger than those in the two earlier independent studies [18,19] (Table 1). Variation was also noted in the regional distribution of the measured children (*p* = 0.000) (Table 1). This is important as it may affect the generalisability of the findings and may not adequately reflect the geographical distribution of target study population in real-world settings.

## 5. Conclusions

In summary, childhood overweight and obesity prevalence in Croatia is slowly starting to see a slowdown in growth rate after years of an increasing trend recorded since the first available data collected in 2003. Hence, Croatia appears to be following the same trend of decelerating overweight prevalence among school-aged children seen in other European countries, albeit with a lag of several years. Whether such a trend will continue is yet to be seen, given that some predictions of future overweight prevalence rates in children and adolescents in Croatia propose drastically worsening rates by the year 2060 [38]. Importantly, this study highlights the regional differences in the overweight prevalence that require a follow-up if such differences are to be understood and acted upon in the form of more precise public health policies focused on health promotion and overweight and obesity prevention from an early age. Lastly, given that childhood overweight and obesity have a considerable negative impact on health in childhood and later in life, and in the context of the demographic decline occurring in Croatia, it is imperative that the complex problem of childhood overweight and obesity is tackled more proactively through multi-sectoral collaboration beyond the scope of healthcare, development of national and inclusion of international overweight and obesity prevention and treatment guidelines, longitudinal data collection and persistent dialogue amongst all the relevant stakeholders that could and should make a difference.

## Figures and Tables

**Table 1:** Characteristics of cross-sectional data points and study participants.

Timepoint	Participation Rate (%)	Participant N (% of Total N in the Study)	Age		Sex		NUTS-2 Region			
			7-Year-Olds N (%)	8-Year-Olds N (%)	Female N (%)	Male N (%)	Participants from City of Zagreb Region (%)	Participants from Pannonian Region (%)	Participants from Adriatic Region (%)	Participants from Northern Region (%)
2003/2004 [18]	91.9%	913 (7.7%)	854 (93.5%)	59 (6.5%)	444 (48.6%)	469 (51.4%)	13.7%	23.5%	43.2%	19.6%
2006–2008 [19]	94.5%	1920 (16.2%)	949 (49.4%)	971 (50.6%)	930 (48.4%)	990 (51.6%)	13.0%	22.1%	42.4%	22.4%
2015/2016 [21]	79.2%	3696 (31.3%)	958 (25.9%)	2738 (74.1%)	1871 (50.6%)	1825 (49.4%)	27.0%	17.1%	23.3%	32.5%
2018/2019 [20]	80.0%	2816 (23.8%)	124 (4.4%)	2692 (95.6%)	1415 (50.2%)	1401 (49.8%)	20.1%	22.0%	24.6%	33.2%
2021/2022 [15]	70.5%	2472 (20.9%)	110 (4.5%)	2362 (95.5%)	1234 (49.9%)	1238 (50.1%)	23.0%	35.3%	18.6%	23.2%
Overall	N/A	11817 (100%)	2995 (25.3%)	8822 (74.7%)	5894 (49.9%)	5923 (50.1%)	21.2%	23.4%	27.3%	28.1%

7-year-olds: ≥7.00 and <8.00 years; 8-year-olds: ≥8.00 and <9.00 years.

**Table 2:** Prevalence rates of overweight (including obesity), obesity and severe obesity in Croatian children aged 7.00–8.99 years, based on the IOTF and WHO growth reference cut-offs.

Timepoint	Overweight Prevalence (%)		Obesity Prevalence (%)		Severe Obesity Prevalence (%)	
	IOTF	WHO	IOTF	WHO	IOTF	WHO
2003/2004	20.6	26.3	7.3	12.1	3.1	3.9
2006–2008	25.3	31.7	8.5	15.0	3.0	3.9
2015/2016	27.1	34.8	8.6	14.2	2.3	3.6
2018/2019	27.2	34.8	9.3	14.7	2.3	3.6
2021/2022	28.0	36.2	8.6	15.2	2.0	3.4

**Table 3:** Risk of overweight (including obesity) in measured children adjusted for sex, time (linear), age (linear), region, based on the IOTF growth reference.

Independent Variable		OR	95% CI	*p*-Value
Time		1.017	1.009–1.026	0.000 *
Sex	Male sex	1.000		
	Female sex	0.956	0.881–1.038	0.284
Age		0.990	0.892–1.099	0.855
NUTS-2 region	City of Zagreb	1.000		
	Pannonian region	1.480	1.303–1.681	0.000 *
	Adriatic region	1.529	1.349–1.732	0.000 *
	Northern region	1.497	1.324–1.692	0.000 *

Abbreviations: OR, odds ratio; CI confidence interval. Significant *p*-value: *p* < 0.05, marked with an asterisk *.

**Table 4:** Risk of overweight (including obesity) by sex adjusted for time (linear), age (linear), region, based on the IOTF growth reference.

Sex	Independent Variable	OR	95% CI	*p*-Value
Female	Time	1.014	1.002–1.026	0.023 *
	Age	1.098	0.947–1.272	0.216
	City of Zagreb	1.000		
	Pannonian region	1.458	1.218–1.746	0.000 *
	Adriatic region	1.440	1.208–1.717	0.000 *
	Northern region	1.388	1.166–1.653	0.000 *
Male	Time	1.021	1.009–1.033	0.001 *
	Age	0.893	0.770–1.036	0.134
	City of Zagreb	1.000		
	Pannonian region	1.496	1.249–1.791	0.000 *
	Adriatic region	1.620	1.357–1.935	0.000 *
	Northern region	1.609	1.354–1.912	0.000 *

Abbreviations: OR, odds ratio; CI confidence interval. Significant *p*-value: *p* < 0.05, marked with an asterisk *.

**Table 5:** Risk of overweight (including obesity) by region adjusted for time (linear), age (linear), sex, based on the IOTF growth reference.

NUTS-2 Region	Independent Variable	OR	95% CI	*p*-Value
City of Zagreb	Time	1.005	0.983–1.027	0.661
	Age	0.860	0.669–1.106	0.240
	Female sex	1.042	0.859–1.263	0.679
	Male sex	1.000		
Pannonian region	Time	1.000	0.983–1.016	0.961
	Age	1.155	0.926–1.441	0.201
	Female sex	1.014	0.859–1.198	0.869
	Male sex	1.000		
Adriatic region	Time	1.028	1.013–1.044	0.000 *
	Age	0.963	0.799–1.159	0.687
	Female sex	0.917	0.786–1.071	0.274
	Male sex	1.000		
Northern region	Time	1.027	1.010–1.044	0.002 *
	Age	0.983	0.808–1.196	0.864
	Female sex	0.898	0.771–1.045	0.163
	Male sex	1.000		

Abbreviations: OR, odds ratio; CI confidence interval. Significant *p*-value: *p* < 0.05, marked with an asterisk *.

## Data Availability

The original contributions presented in this study are included in the article/Supplementary Materials. Further inquiries can be directed to the corresponding author.

## Supplementary Materials

The following supporting information can be downloaded at: https://www.mdpi.com/article/10.3390/children12101299/s1. Table S1: Prevalence of overweight including obesity, obesity and severe obesity by sex in Croatian children aged 7.00–8.99 years, based on the IOTF and WHO growth reference cut-offs; Table S2: Prevalence of overweight including obesity, obesity and severe obesity by region in Croatian children aged 7.00–8.99 years, based on the IOTF and WHO growth reference cut-offs; Table S3: Obesity risk adjusted for sex, time (linear), age (linear), region, based on the IOTF growth reference; Table S4: Obesity risk by sex adjusted for time (linear), age (linear), region, based on the IOTF growth reference; Table S5: Obesity risk by region adjusted for time (linear), age (linear), sex, based on the IOTF growth reference.

## Abbreviations

The following abbreviations are used in this manuscript:

BMI	Body mass index
CI	Confidence interval
COSI	Childhood Obesity Surveillance Initiative
CroCOSI	Childhood Obesity Surveillance Initiative, Croatia
EHIS	European Health Interview Survey
EU	European Union
GDP	Gross domestic product
IOTF	International Obesity Task Force
NUTS	Nomenclature of Territorial Units for Statistics
OR	Odds ratio
SES	Socio-economic status
SPSS	Statistical Package for the Social Sciences
WHO	World Health Organization
