# Supplementary material for: Trends in the Prevalence of Overweight, Obesity and Severe Obesity in Primary School Children in Croatia from 2003 to 2022
Source: Children (Basel). 2025 Sep 25;12(10):1299. doi: 10.3390/children12101299 (PMC12564036; doi:10.3390/children12101299)
Supplement: Supplementary file 1 [file children-12-01299-s001.zip › children-3893132-supplementary.pdf]

children

MDPI

Original research article:
Trends in the Prevalence of Overweight, Obesity and Severe Obesity in Primary School Children in Croatia from 2003 to 2022

Supplementary materials

Timepoint	Sex	Overweight prevalence (%)		Obesity prevalence (%)		Severe obesity prevalence (%)	
		IOTF	WHO	IOTF	WHO	IOTF	WHO
2003/2004	Girls	18.9	22.3	6.5	9.0	2.7	2.0
	Boys	22.2	30.0	8.1	15.0	3.4	5.6
2006-2008	Girls	25.6	29.9	8.5	13.1	2.7	2.5
	Boys	25.1	33.3	8.5	16.9	3.3	5.2
2015/2016	Girls	26.0	31.0	6.9	10.5	2.0	1.7
	Boys	28.1	38.8	10.2	18.0	2.7	5.6
2018/2019	Girls	27.3	32.7	8.5	11.7	1.9	1.7
	Boys	27.0	37.0	10.1	17.7	2.6	5.5
2021/2022	Girls	27.6	33.7	7.8	12.1	1.1	1.1
	Boys	28.3	38.6	9.4	18.2	2.8	5.8

Supplementary table S1. Prevalence of overweight including obesity, obesity and severe obesity by sex in Croatian children aged 7.00-8.99 years, based on the IOTF and WHO growth reference cut-offs.

Timepoint	NUTS-2 region	Overweight prevalence (%)		Obesity prevalence (%)		Severe obesity prevalence (%)	
		IOTF	WHO	IOTF	WHO	IOTF	WHO
2003/2004	City of Zagreb	12.8	20.0	2.4	8.0	0.8	0.8
	Pannonian	21.4	26.5	8.8	12.1	2.8	5.1
	Adriatic	23.1	28.6	8.4	13.5	4.3	5.1
	Northern	19.6	25.4	6.7	11.9	2.2	1.7
2006-2008	City of Zagreb	26.4	35.2	6.4	15.2	1.6	3.6
	Pannonian	28.7	36.2	11.1	17.5	5.4	5.7
	Adriatic	23.8	29.4	8.1	14.6	2.5	3.8
	Northern	24.1	29.4	7.9	13.3	2.6	2.3
2015/2016	City of Zagreb	20.9	29.1	6.6	11.1	1.8	2.9
	Pannonian	31.2	39.5	11.1	16.8	3.2	4.4
	Adriatic	28.5	35.4	8.7	14.8	2.2	3.7
	Northern	29.0	36.8	8.8	15.0	2.4	3.7
2018/2019	City of Zagreb	19.9	29.6	6.0	8.6	0.5	1.4
	Pannonian	25.2	30.4	10.5	14.9	2.9	4.4
	Adriatic	33.2	40.2	12.6	20.7	4.6	6.1
	Northern	28.4	37.0	8.0	13.8	1.2	2.6
2021/2022	City of Zagreb	21.1	29.0	5.8	10.0	0.7	1.2
	Pannonian	29.0	38.5	7.2	15.0	1.8	3.7
	Adriatic	29.4	36.0	10.9	15.7	2.4	3.1
	Northern	31.9	39.7	11.5	20.0	3.1	5.4

Supplementary table S2. Prevalence of overweight including obesity, obesity and severe obesity by region in Croatian children aged 7.00-8.99 years, based on the IOTF and WHO growth reference cut-offs.

Independent variable	OR	95% CI	p-value
Time	1.015	1.002-1.029	0.026*
Male sex	1.000		
Female sex	0.787	0.692-0.896	0.000*
Age	0.906	0.768-1.068	0.239
City of Zagreb	1.000		
Pannonian region	1.641	1.333-2.109	0.000*
Adriatic region	1.711	1.396-2.098	0.000*
Northern region	1.504	1.227-1.843	0.000*

Supplementary table S3. Obesity risk adjusted for sex, time (linear), age (linear), region, based on the IOTF growth reference.

Abbreviations: OR, odds ratio; CI confidence interval

Significant p-value: $p < 0.05$, marked with an asterisk*

Sex	Independent variable	OR	95% CI	p-value
Female	Time	1.001	0.982-1.021	0.901
	Age	1.172	0.921-1.021	0.197
	City of Zagreb	1.000		
	Pannonian region	1.685	1.236-2.297	0.001*
	Adriatic region	1.835	1.359-2.479	0.000*
	Northern region	1.360	0.997-1.855	0.052
Male	Time	1.028	1.009-1.047	0.003*
	Age	0.722	0.576-0.905	0.005*
	City of Zagreb	1.000		
	Pannonian region	1.591	1.203-2.104	0.001*
	Adriatic region	1.599	1.212-2.111	0.001*
	Northern region	1.613	1.231-2.112	0.001*

Supplementary table S4. Obesity risk by sex adjusted for time (linear), age (linear), region, based on the IOTF growth reference.

Abbreviations: OR, odds ratio; CI confidence interval

Significant p-value: $p < 0.05$, marked with an asterisk*

NUTS-2 region	Independent variable	OR	95% CI	p-value
City of Zagreb	Time	1.019	0.981-1.059	0.320
	Age	0.820	0.532-1.266	0.372
	Female sex	0.774	0.556-1.077	0.129
	Male sex	1.000		
Pannonian region	Time	0.978	0.954-1.003	0.078
	Age	1.197	0.860-1.666	0.287
	Female sex	0.826	0.640-1.066	0.142
	Male sex	1.000		
Adriatic region	Time	1.037	1.013-1.061	0.002*
	Age	0.785	0.589-1.046	0.098
	Female sex	0.900	0.712-1.137	0.377
	Male sex	1.000		
Northern region	Time	1.025	0.997-1.052	0.077
	Age	0.882	0.645-1.206	0.432
	Female sex	0.656	0.513-0.837	0.001*
	Male sex	1.000		

Supplementary table S5. Obesity risk by region adjusted for time (linear), age (linear), sex, based on the IOTF growth reference.

Abbreviations: OR, odds ratio; CI confidence interval

Significant p-value: $p < 0.05$, marked with an asterisk*
